# Is an abdominal cerclage indicated with a cervical myomectomy? A case report

**DOI:** 10.1016/j.crwh.2021.e00345

**Published:** 2021-07-21

**Authors:** Vanille Simon, Marie-Ève Bergeron, André Lamarre, Philippe Laberge, Sarah Maheux-Lacroix

**Affiliations:** a Department of Obstetrics and Gynecology, CHU de Québec- Université Laval, 2705 boulevard Laurier, Quebec City, G1V 4G2 QC, Canada; b Department of Interventional Radiology, CHU de Québec- Université Laval, 2705 boulevard Laurier, Quebec City, G1V 4G2 QC, Canada

**Keywords:** Cervical leiomyoma, Cervical cerclage, Laparoscopic myomectomy, Uterine artery embolization, Case report

## Abstract

Introduction: Cervical myomectomy can compromise cervical integrity and the risk of subsequent cervical incompetence is unclear. In this case report, the literature on cervical myomectomies is reviewed as well as that on the potential benefits of cervical cerclage. Case presentation: A 30-year-old woman, nulligravida, with a 12 cm cervical leiomyoma consulted for heavy menstrual bleeding and pelvic pain. After failure of multiple medical therapies, a laparoscopic cervical myomectomy was successfully performed after pre-operative uterine artery embolization using absorbable gelatin sponges to reduce surgical blood loss. Discussion: A concomitant laparoscopic cerclage was achieved in order to prevent cervical incompetence, given that the full thickness of the anterior cervix was penetrated during the myomectomy.

## Highlights

• Laparoscopic myomectomy of large cervical leiomyomas is feasible with minimal blood loss with proper preparation.
• Adjuvant preoperative uterine artery embolization is an alternative to ligation when the retroperitoneum is inaccessible.
• Concomitant laparoscopic cerclage is reasonable to prevent cervical insufficiency and does not alter the mode of delivery.

## 1 Introduction

Uterine leiomyomas are frequent benign uterine tumors, affecting nearly 80% of women by age 50 [1]. However, those arising from the cervix account for less than 5% of leiomyomas [1]. They often have a complex vascularization and are responsible for anatomic distortion [1,2]. Therefore, laparoscopic cervical myomectomy represents a surgical challenge, with a risk of bleeding and injury to surrounding organs. The procedure can also cause significant damage to the cervix, but the risk of cervical incompetence remains unknown. In fact, the literature on cervical leiomyomas management is poor and mainly consists of a few case reports and retrospective studies [1,3,4].

We present a case of minimally invasive surgical management of a large cervical leiomyoma in a woman who wished to preserve fertility and a review of the literature on the role of a concomitant cervical cerclage.

## 2 Case Presentation

**Fig. 1:**
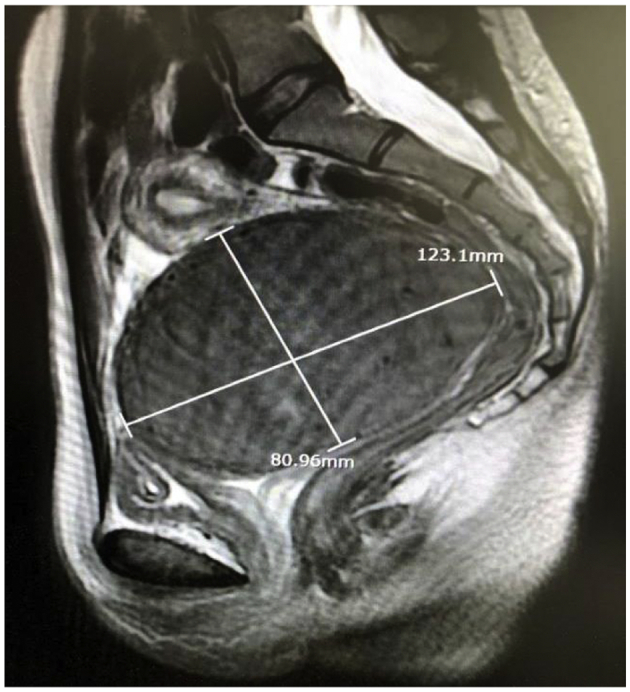
Pelvic magnetic resonance imaging, in sagittal T2-weighted sequence. A large 12 cm leiomyoma of left lateral cervical sidewall, FIGO 8, filling the pelvis.

A 30-year-old woman consulted for severe heavy menstrual bleeding and pelvic pain. She was in good health and had never been sexually active or pregnant. Upon vaginal examination, a large and solid mass with little overall mobility was palpable at 1 cm of the vaginal introitus and was filling the pelvis. An endometrial biopsy could not be performed as the cervical os was not identifiable during physical examination but was deemed non-essential given the low risk of malignancy and reassuring imaging. Ultrasonography and magnetic resonance imaging (MRI) revealed a large cervical leiomyoma of 12 × 8 × 10 cm with moderate right ureterohydronephrosis (Fig. 1).

Several medical therapies had already been sequentially attempted: tranexamic acid 4000 mg/day, ulipristal acetate 5 mg/day for 3 months, and seven injections of leuprolide acetate 11.25 mg with add-back. Although hydronephrosis had resolved, bleeding remained severe, with anemia (hemoglobin: 80 g/L), and a uterine artery embolization with non-absorbable hydrogel microspheres of 500 μm (Embozene™, Boston Scientific) failed to control symptoms. On MRI, a 11x8x10 cm leiomyoma of the left lateral cervical sidewall was still present. Its appearance was compatible with a benign leiomyoma and the LDH level was normal (LDH: 150 U/L).

After referral to a specialist in minimally invasive gynecology, a myomectomy was decided. The patient was informed about the surgical risks (hemorrhage, blood transfusion, conversion to laparotomy, emergency hysterectomy, infertility, thrombosis, infection, injury to surrounding organs) and that a caesarean delivery would likely be required for any subsequent pregnancy. Consent was obtained to perform a concomitant prophylactic cerclage in case of significant damage to the cervix. Continued leuprolide acetate and iron supplementation corrected anemia before surgery.

### 2.1 Reduction of Bleeding

On surgery day, all members of the surgical and anesthesic teams were aware of the potential risk of significant bleeding and conversion to laparotomy. As surgeons anticipated that the size and location of the leiomyoma would prevent uterine artery ligation with temporary clips, adjuvant uterine artery embolization was performed two hours before the surgery using absorbable gelatin sponge particles (GelFoam™, Pfizer). In addition, we administered 800 μg of intrarectal misoprostol at induction, 1000 mg of intravenous tranexamic acid at skin incision and sub-serosal injection of vasopressine (10UI diluted in 100 mL of saline solution). Blood products were available.

### 2.2 Myomectomy

**Fig. 2:**
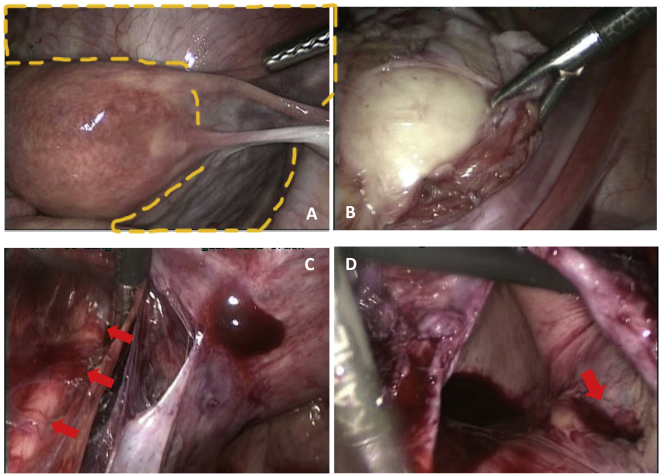
Laparoscopic cervical myomectomy. **A**. Intra-operative laparoscopic view of a large cervical leiomyoma filling the pelvis (yellow dashed line). **B**. The correct plan of dissection is identified and the myomectomy is completed by enucleation, applying traction-countertraction using a long-pitch corkscrew. Here, the grasper is pushing downward the leiomyoma pseudocapsule. The leiomyoma was exteriorized using power morcellation (LiNA Xcise™, LiNA) at the end of the surgery. **C**. Bilateral ureterolysis is done (red arrow = left ureter). **D**. The anterior vaginal wall is opened allowing identification of the external cervical os (red arrow). (For interpretation of the references to colour in this figure legend, the reader is referred to the web version of this article.)

Myomectomy was performed using the standard 4-ports laparoscopic technique (diamond configuration). A large intra-mural cervical leiomyoma occupied the entire pelvis (Fig. 2A). The retroperitoneum was open, and ureters identified at the level of the pelvic brim. Using the monopolar scissors, the bladder was dissected down. A central and anterior cervical incision was performed (Fig. 2B). Once the myomectomy was initiated, it was possible to pull the leiomyoma anteriorly with a corkscrew and complete the bilateral ureterolysis down to the uterine arteries (Fig. 2C). The myomectomy was then completed, applying traction-countertraction, with ureters, bladder, and bowel under view control.

### 2.3 Cervical Cerclage

**Fig. 3:**
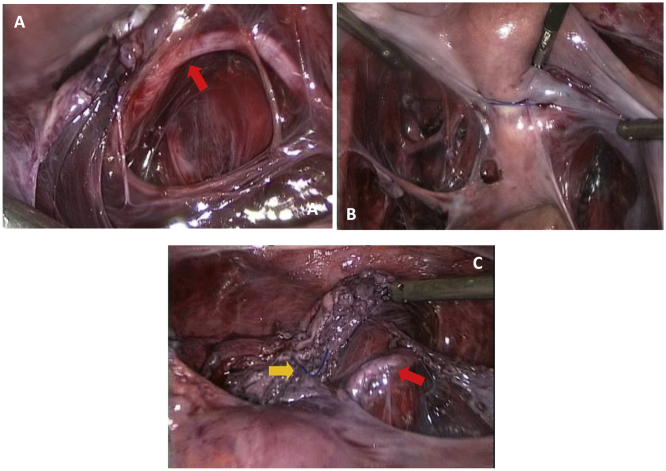
Concomitant laparoscopic cerclage. **A**. Uterine arteries are skeletonized (red arrow = left uterine artery). **B**. Two loops of non-absorbable 0-prolene suture are placed around the cervix at the height of the cervico-isthmic junction, just above the insertion of the utero-sacral ligaments, by passing the needle medially to the uterine vessels. **C**. A secure knot is tied anteriorly (yellow arrow). The bladder has already been dissected down at the beginning of surgery. Purple-coloured tissues are remaining fibers of the cervix after myomectomy. The right uterine artery is visible on the right (red arrow). (For interpretation of the references to colour in this figure legend, the reader is referred to the web version of this article.)

The anterior vaginal wall was entered inadvertently during the myomectomy, allowing identification of the external cervical os (Fig. 2D). Since the full thickness of the anterior cervix was penetrated, a cerclage was performed. Broad ligaments were fenestrated on both sides and uterine arteries skeletonized at the isthmus of the uterus (Fig. 3A). Two loops of non-absorbable suture (0-Prolene, Ethicon) were placed around the cervix at the height of the cervico-isthmic junction (Fig. 3B) by passing the needle medially to the uterine vessels. The level of the cerclage was determined by identifying the uterosacral ligaments posteriorly and the vaginal fornix anteriorly.

The surgery lasted 170 min, intra-operative blood loss was 150 mL and post-operative hemoglobin was 109 g/L. A benign leiomyoma was confirmed at histopathology and weighted 444 g. The patient was hospitalized for less than 24 h, had no post-operative complication and her menstrual flow was back to normal 10 weeks after surgery.

## 3 Discussion

Abdominal cerclage, by laparotomy or laparoscopy, is typically offered to women who have had at least two prior failed vaginal cerclages, with neonatal survival rates between 71 and 100% [5]. It is also performed for women undergoing trachelectomies for cervical cancer to reduce pregnancy loss [5]. Data is lacking about the risk of cervical incompetence following cervical myomectomy but damage to the cervix can be severe, especially with large and deep cervical leiomyomas. As potential obstetrical complications are serious, including neonatal death, it seems reasonable to offer a concomitant cerclage when the integrity of the cervix is severely compromised by the myomectomy, especially as a caesarean delivery is already required [6].

Intra-operative hemorrhage is also a significant concern during myomectomy, especially with cervical myomectomies [2]. In fact, leiomyomas of the cervix are often vascularized by both vaginal and uterine arteries as well as complex network of neovascularization [4]. Although intra-operative temporary or permanent occlusion of uterine arteries with clips, bulldog clamps, ligatures or tourniquet are good options to reduce intra-operative bleeding [2], these techniques may not be suitable in cases of large cervical leiomyomas due to limited access to the retroperitoneum. Adjuvant pre-operative uterine artery embolization is an alternative [7]. Compared to permanent inert polyvinyl alcohol or tris-acryl gelatin microparticles, pledgets of gelatin sponge particles (GelFoam) are completely dissolved in vivo within 7 to 21 days, representing an advantage for women who want to preserve fertility.

This case report presents an original reflection on role of a cerclage in certain cases of cervical myomectomy. Evidence is lacking and more studies are needed to better evaluate the rates of fetal loss following a cervical myomectomy and whether an abdominal cerclage could securely prevent adverse outcomes.

### 3.1 Conclusion

Cervical myomectomy can be performed by laparoscopy while combining several interventions to reduce blood loss. When the integrity of the cervix is significantly damaged by the myomectomy, placement of an abdominal cerclage should be considered to prevent cervical incompetence.
